# Nitrification Inhibitor 3,4-Dimethylpyrazole Phosphate Application During the Later Stage of Apple Fruit Expansion Regulates Soil Mineral Nitrogen and Tree Carbon–Nitrogen Nutrition, and Improves Fruit Quality

**DOI:** 10.3389/fpls.2020.00764

**Published:** 2020-06-03

**Authors:** Fen Wang, Xinxiang Xu, Zhihang Jia, Xin Hou, Qian Chen, Jianchuan Sha, Zhaoxia Liu, Zhanling Zhu, Yuanmao Jiang, Shunfeng Ge

**Affiliations:** State Key Laboratory of Crop Biology, College of Horticulture Science and Engineering, Shandong Agricultural University, Tai’an, China

**Keywords:** apple, DMPP, mineral nitrogen, ^15^N, ^13^C, fruit quality

## Abstract

In order to solve the problems of nitrogen (N) losses and fruit quality degradation caused by excessive N fertilizer application, different dosages of the nitrification inhibitor, 3,4-dimethylpyrazole phosphate (DMPP) (0, 0.5, 1, 2, and 4 mg kg^–1^ soil), were applied during the later stage of ‘Red Fuji’ apple (*Malus domestica* Borkh.) fruit expansion in 2017 and 2018. The effects of DMPP on soil N transformation, carbon (C)–N nutrition of tree, and fruit quality were investigated. Results revealed that DMPP decreased the abundance of ammonia-oxidizing bacteria (AOB) *amoA* gene, increased the retention of NH_4_^+^-N, and decreased NO_3_^–^-N concentration and its vertical migration in soil. DMPP reduced ^15^N loss rates and increased ^15^N residual and recovery rates compared to the control. ^13^C and ^15^N double isotope labeling results revealed that DMPP reduced the capacity of ^15^N absorption and regulation in fruits, decreased ^15^N accumulation in fruits and whole plant, and increased the distribution of ^13^C from vegetative organs to fruits. DMPP increased fruit anthocyanin and soluble sugar contents, and had no significant effect on fruit yield. The comprehensive analysis revealed that the application of 1 mg DMPP kg^–1^ soil during the later stage of fruit expansion effectively reduced losses due to N and alleviated quality degradation caused by excessive N fertilizer application.

## Introduction

China possesses the largest apple cultivation area and production in the world (FAOSTAT, 2018). Unilateral pursuit of high yields and large fruits by fruit farmers, the excessive application of nitrogen (N) fertilizer has become a common problem in China. At present, the amount of N fertilizer application in apple orchards has reached 600 to 800 kg ha^–1^, which far exceeds the demands of plants (Ge et al., 2015; Zhu et al., 2018). Farmers have applied more N fertilizer during the early stages of the growing season in order to meet the growth and development needs of apple trees, although the utilization rate of N fertilizer in apple trees is generally low (5.2–31.3%) (Liu et al., 2010; Ding et al., 2017; Wang et al., 2020b). Most N fertilizer that is not absorbed has been left in the soil profile as inorganic N or organic combination forms and is integrated into the soil N pool (Ju, 2014).

During the later stage of apple fruit expansion, high temperature and rainy weather would lead to the mineralization of organic N in soil and to produce a large amount of ammonium N. And ammonium N can be easily transformed into nitrate N through nitrification (Dessureault-Rompré et al., 2010; Guntiñas et al., 2012). Nitrate N pollutes surface water and groundwater through surface runoff and leaching losses, as well as the atmosphere through denitrification (Vinzent et al., 2018; Wen et al., 2019). Additionally, nitrate N is more easily absorbed by apple trees than ammonium N (Li et al., 2013). Large amount of absorbed nitrate N would affect the carbon (C)–N balance of trees and result in excessive N in apple fruits. The imbalance of C and N in trees is not conducive to the flow of photosynthetic to fruits, and high N contents of fruits negatively affect fruits color, soluble solids, and other quality indicators (Wang et al., 2017; Wang X. F. et al., 2018; An et al., 2018; Zhang et al., 2020). Therefore, applying exogenous substances to inhibit soil nitrification is important for simultaneously controlling agricultural N pollution and improving fruit quality.

Nitrification inhibitors are widely used to delay the bacterial oxidation of NH_4_^+^ to nitrite (NO_2_^–^) by suppressing ammonia monooxygenase (AMO) activities in soil (Chen et al., 2010; Bell et al., 2016; Gilsanz et al., 2016). Recent researches about *amoA* gene, the gene that encodes the first subunit of AMO enzyme, revealed that ammonia-oxidizing archaea (AOA) and ammonia-oxidizing bacteria (AOB) play a major role in soil nitrification (Chen et al., 2015, 2019). Nitrification inhibitors decrease N losses by reducing NO_3_^–^ leaching through the retention of N in low mobility forms (e.g., NH_4_^+^) and by decreasing N_2_O emissions through the reduction of NO_3_^–^ concentrations for the denitrification process (Zhu et al., 2015; Friedl et al., 2017; Ni et al., 2018; Wang et al., 2020c). The new nitrification inhibitor, 3,4-dimethylpyrazole phosphate (DMPP), has many advantages, including application in small dosages, a long aging time, and non-toxicity, and it does not pollute the environment (Zerulla et al., 2001; Macadam et al., 2003). Therefore, DMPP has the potential to be a good nitrification inhibitor in agricultural management practices. A previous study found that DMPP decreased gross soil autotrophic nitrification rates and reduced gross mineralization rates through feedback regulation (Zhu et al., 2019). DMPP application reduced the risk of nitrate leaching and N losses due to denitrification and did not increase NH_3_ volatilization (Zerulla et al., 2001; Li et al., 2008). Yin et al. (2012) also found that the inhibition effects and associated time of DMPP on nitrification increased as the DMPP dosage increased, but when the dosage was >2%, the enhancement of the inhibition effect was no longer obvious.

Currently, researches on nitrification inhibitor DMPP are mainly focused on soil N transformation and N losses (Chen et al., 2019; Li et al., 2020b; Mateo-Marin et al., 2020; Vilarrasa-Nogue et al., 2020). Moreover, its effects on fruit quality and its application in apple orchards are rarely reported. Therefore, in this study, the effects of DMPP on apple soil mineral N, C–N nutrition of tree, and fruit quality were investigated to provide a reference for reducing N losses and improving fruit quality.

## Materials and Methods

### Experimental Site and Materials

**FIGURE 1:**
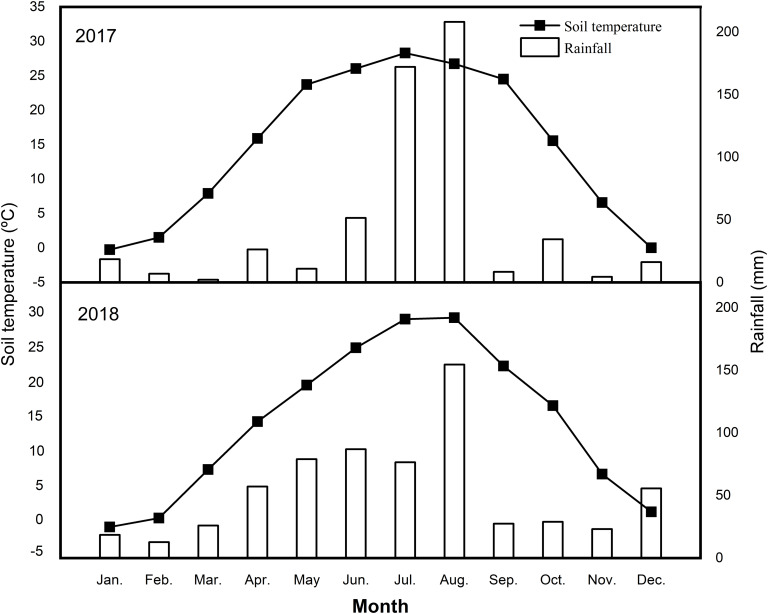
Mean monthly rainfall and soil temperature at 5 cm depth in the years of 2017 and 2018.

This study was performed from 2017 to 2018 in an apple orchard located in Laishan, Yantai City, Shandong Province, Northeast China (121°43′00″E, 37°50′47″N). The climate is semi-humid with an annual average precipitation of 672.5 mm, of which nearly 70% occurs from June to September. The mean monthly rainfall and soil temperature at the 5 cm soil depth during the study are presented in Figure 1.

Trees were planted in 2012 in rows spaced 1.5 m apart with 4 m between rows and were trained as a slender spindle. The commercially important ‘Red Fuji’ apple cultivar (*Malus* × *domestica* Borkh.) was grafted on the dwarfing interstock, M.26, then grafted on *M. hupehensis* Rehd. rootstocks (‘Red Fuji’/M.26/*M. hupehensis* Rehd.). The basic physicochemical properties of the soil are presented in Table 1.

**TABLE 1:** Basic physicochemical properties of the experimental soil.

Year	Soil layer (cm)	Organic matter (g kg^–1^)	Alkali-hydrolyzable N (mg kg^–1^)	Available P (mg kg^–1^)	Available K (mg kg^–1^)	Bulk density (g cm^–3^)
2017	0–20	18.05	69.67	38.19	219.13	1.13
	20–40	15.62	51.36	27.34	203.05	1.27
	40–60	13.23	41.89	15.42	191.62	1.35
2018	0–20	18.22	68.46	40.03	214.69	1.13
	20–40	16.11	52.93	25.45	208.94	1.28
	40–60	13.20	43.85	16.79	189.27	1.38

### Experimental Design and Sampling

In this study, 30 trees with similar growth potential were selected and treated with repeated applications of five treatments in 2017 and 2018. The treatments included Treatment 1: Control (0 mg DMPP kg^–1^ soil; water as control), Treatment 2: T_1_ (0.5 mg DMPP kg^–1^ soil), Treatment 3: T_2_ (1 mg DMPP kg^–1^ soil), Treatment 4: T_3_ (2 mg DMPP kg^–1^ soil), and Treatment 5: T_4_ (4 mg DMPP kg^–1^ soil). In each treatment, 0, 0.42, 0.85, 1.70, and 3.39 g DMPP plant^–1^ was applied. The dosage to each tree was calculated by the mass of soil at the 60 cm depth within the projected area of the tree crown. Treatments were conducted during the later stage of fruit expansion (105 days after blooming). The application method was as follows: DMPP was dissolved in 4 L of water and evenly distributed in 12 spots within the projected area occupied by the crown of a single tree, then DMPP was applied to 12 spots using a fertilizer gun at the 10, 30, and 50 cm soil depths.

Based on isotope labeling, each treatment was divided into two groups with three replicates per group and two trees per replicate as follows. Group 1: 340 g normal urea (CO(NH_2_)_2_), 210 g ammonium phosphate ((NH_4_)_2_HPO_4_), and 120 g potassium sulfate (K_2_SO_4_) were applied to each tree as the non-labeled group, where 50% of the fertilizer was applied at the germination stage and 50% as fruit setting fertilizer (40 days after blooming). Group 2: 10 g ^15^N-urea (CO(^15^NH_2_)_2_ produced by the Shanghai Research Institute of Chemical Industry, 10.22% abundance), 330 g normal CO(NH_2_)_2_, 210 g (NH_4_)_2_HPO_4_, and 120 g K_2_SO_4_ were applied to each tree as the labeled group, where 50% of the fertilizer was applied at the germination stage and 50% as fruit setting fertilizer (40 days after blooming). Subsequently, ^13^C pulse labeling was performed in a labeling chamber 182 days after blooming in 2017 and 2018. Fertilizer was applied by digging a circular trench with a 30-cm radius around each tree and a width and depth of 20 cm. The growth conditions, cultivation, and management of all treatments were consistent across treatments and years.

Soil samples were obtained 20, 40, 60, and 80 days after DMPP application. The soil sampling method was as follows: 12 sampling points were evenly distributed throughout the projected area of the tree canopy occupied by a single tree; the soil sample depths of 0–20, 20–40, 40–60, 60–80, and 80–100 cm were retrieved in the vertical direction of each soil extraction point; 12 soil samples per layer were evenly mixed as one replicate. After collection, soil samples were immediately transferred to the laboratory to determine gene abundance, mineral N (NH_4_^+^-N and NO_3_^–^-N) contents and ^15^N residues (calculated to the 60 cm depth). All plants were subjected to destructive sampling at the fruit maturity stage (185 days after blooming). Fruits were selected from four directions in the middle of the outer part of the crown with 12 fruits in each tree. Fruit peels and flesh were immediately frozen in liquid N and stored at −80°C for further analysis.

### ^13^C Labeling Method

The ^13^C labeling method used in this study was previously described by Wang et al. (2020a). Each tree of Group 2 was individually covered and sealed by a labeling chamber, which was composed of 0.1-mm-thick Mylar plastic bags and brackets. Put fans and beaker contained with 10 g of Ba^13^CO_3_ (^13^C abundance is 98%) into the labeling room, turned on the fans and sealed the labeling chamber. Labeling work started at 8:00 am (182 days after blooming). 1 mL of hydrochloric acid (1 mol L^–1^) was injected into the beaker with a syringe every 0.5 h in order to maintain the concentration of ^13^CO_2_, ^13^C labeling process lasted for 4 h. In order to prevent excessive temperature during the labeling process, appropriate amount of ice bag was added to the bottom of labeling chamber to control the temperature in the range of 28–37°C. All trees were destructively sampled after 72 h (185 days after blooming). The trees of Group 1 were destructively sampled and used as a blank for ^13^C labeling (natural abundance of ^13^C).

### DNA Extraction and Quantitative PCR of AOA and AOB *amoA* Genes

The 0–60 cm soil sample of each tree is mixed as one replicate to measure the abundance of AOA and AOB *amoA* genes. DNA was extracted using the FastDNA SPIN Kit for soil (Bio101, Vista, CA, United States) according to the manufacturer’s instructions. Real-time quantitative PCR of *amoA* genes was according to Chen et al. (2019). The details of primers, reaction mixture compositions, and thermal cycling conditions are listed in Supplementary Table S1.

### Concentrations of Soil Ammonium and Nitrate

Soil ammonium and nitrate were extracted with 0.01 M KCl and analyzed using a San^++^ continuous flow analyzer (Skalar Analytical, Breda, Netherlands) (Duan et al., 2015).

### Ammonia Volatilization

Ammonia volatilization was measured every 10 days after DMPP application. Twelve ammonia volatilization measurement points were evenly distributed in each tree disk. The average of 12 results was used as one replicate. Ammonia volatilization was measured using the ventilation method (Li et al., 2020a). A PVC collection tube (0.20 m diameter, 0.25 m height) was inserted into the soil at a depth of 0.05 m with a phosphoglycerol-soaked sponge placed inside as an absorbent, which was collected (and replaced) daily (10:00 am) throughout the experiment period. The phosphoglycerol-soaked sponges bearing the collected samples were transported to the laboratory and immediately immersed in 500 mL 1.0 mol L^–1^ KCl solution in 1 L polyethylene bottles. Bottles were sealed and shaken at 200 rpm for 1 h on a reciprocating shaker. The NH_4_^+^-N concentrations of the extracted solutions from each bottle were measured by colorimetry (λ = 630 nm) using a UV-VIS spectrophotometer (Unico, Shanghai, China). The NH_3_ volatilization rates were calculated as follows: *R*_AV_ = *M*/(*A* × *D*) × 10^–2^, where *R*_AV_ is the NH_3_ volatilization rate (kg N ha^–1^ d^–1^), *M* is the amount of NH_3_-N collected in the sponge (mg), which is equal to the NH_4_^+^-N contents of the extracted solutions, *A* is the cross-sectional area of the sponge (m^2^), and *D* is the interval of sample collection (d).

### Contents of ^15^N and ^13^C

The whole plant samples were divided into fruits, leaves, annual branches, perennial branches, trunk, and roots. The samples were heated at 105°C for 30 min and then dried at 80°C, followed with homogenization by an electric grinder and filtration with a 0.25 mm mesh screen (Liu et al., 2017). The samples of Group 2 were used to determine the abundance of ^15^N and ^13^C and the content of N, and those of Group 1 were used to determine the natural abundance of ^13^C as a blank control of the corresponding organs of Group 2. The content of N was determined by the Kjeldahl method (Wang et al., 2019), and the abundance of ^15^N was measured with a ZHT-03 mass spectrometer made in the Beijing Analytical Instrument Factory (Chinese Academy of Agricultural Sciences). The abundance of ^13^C was measured with a DELTAV^plus^XP advantage isotope ratio mass spectrometer and analyzed by the China Academy of Forestry Sciences Stable Isotope Laboratory. Three replicates were conducted for each treatment.

Calculation of ^15^N

Ndff(%)=abundance⁢of⁢N15⁢in⁢plant-natural⁢abundance⁢of⁢N15abundance⁢of⁢N15⁢in⁢fertilizer-natural⁢abundance⁢of⁢N15×100%

N15utilizationrate(%)=Ndff×total⁢N⁢of⁢organs⁢(g)N15⁢fertilization⁢(g)×100%

N15residualrate(%)=N15⁢residue⁢in⁢soil⁢(g)N15⁢fertilization⁢(g)×100%

N15lossrate(%)=100%-N15utilizationrate(%)-N15residualrate(%)

N15recoveryrate(%)=100%-N15lossrate(%)

Calculation of ^13^C

AbundanceofC13:F(%)i=(δ⁢C13+1000)×RPBD(δ⁢C13+1000)×RPBD+1000×100%

R(standardratioofcarbonisotope)PBD=0.0112372

$$\mathrm{Carbon}\mathrm{content}\mathrm{of}\mathrm{each}\mathrm{organ}:<\mathrm{cps}:\mathrm{it}>C</\mathrm{cps}:\mathrm{it}>{}_{i}$$

=amountofdrymatter(g)×totalcarboncontent(%)

ContentofC13ofeachorgan:C13(mg)i=Ci×(Fi-Fnl)100×1000

F*_*nl*_*: no ^13^C labeling, natural abundance of ^13^C of each organ

C13distributionrate:C13(%)=Ci13Cnet⁢absorption13×100%(Wang et al.,2020a).

### Fruit Quality

The total anthocyanin content of apple peels was measured according to (Sun et al., 2019) with minor modifications. Each sample (0.5 g) was ground to a powder in liquid N and incubated in 5 mL 1% (v/v) HCl-methanol for 24 h at 4°C in total darkness. After centrifugation, KCl and NaAc buffers were added to the supernatant aliquots, which were mixed and incubated for 20 min at 4°C in total darkness. Solutions were centrifuged at 8000 rcf (× *g*) for 15 min. The absorbance of the supernatant was measured using a UV-2450 spectrophotometer (Shimadzu, Kyoto, Japan) at 510 and 700 nm (i.e., OD_510_ and OD_700_).

The content of soluble sugars was measured using Anthrone colorimetry (Liu et al., 2018). Samples were placed in a test tube, to which 5 mL distilled water was added and mixed after cutting samples into pieces. After 30 min boiling in a water bath, the supernatant was collected. This step was repeated twice, and the volume of the solution was adjusted to 10 mL using distilled water. The absorbance of the solution was determined at 630 nm after adding sulfuric acid and anthrone. The contents of titratable acid were measured by the NaOH titration method (Wang X. et al., 2018). Each treatment had a total of three replicates.

### Statistical Analysis

All the graphs were plotted by Origin 8.0 (OriginLab Corporation, Northampton, MA, United States). Data were analyzed with the IBM SPSS Statistics for Windows Version 19.0 (IBM Corporation, Armonk, NY, United States) by using one-way factorial analysis of variance (ANOVA). In all cases, differences were considered significant at a probability level of *P* < 0.05.

## Results

### Soil N Transformation

#### Abundance of AOA and AOB *amoA* Genes

**FIGURE 2:**
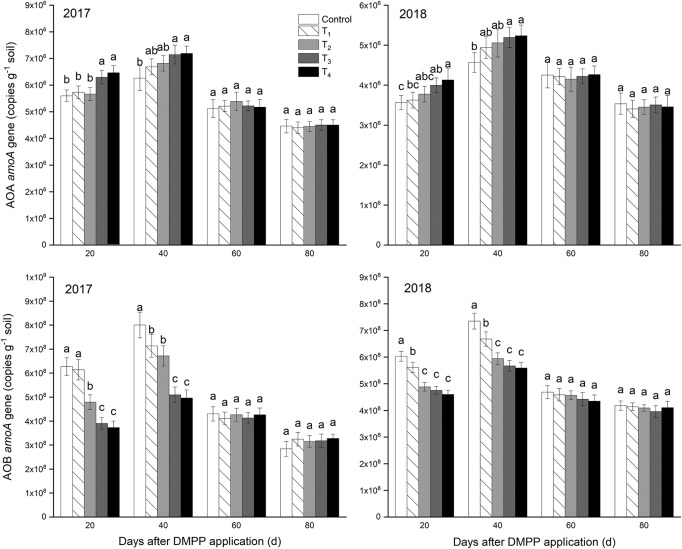
Abundances of *amoA* genes of ammonia-oxidizing archaea (AOA) and bacteria (AOB). The vertical bar indicates the standard deviation of three replications. Different letters indicate statistically significant differences (*P* < 0.05).

The copy numbers of AOA and AOB were assessed via qPCR of their respective *amoA* genes in Figure 2. In general, the abundance of AOB *amoA* gene was higher (2.84 × 10^8^ to 8.01 × 10^8^) than that of AOA *amoA* gene (3.41 × 10^6^ to 7.19 × 10^6^). Over time, the abundance of AOA and AOB *amoA* genes increased first and then decreased, and reached the highest at 40 days. The AOA *amoA* gene abundance increased as the DMPP dosage increased at 20 days and 40 days. DMPP decreased AOB *amoA* gene abundance in different degrees compared to the control at 20 and 40 days, and decreased as the DMPP dosage increased. After 60 days, DMPP had no significant effect on the abundance of AOA and AOB *amoA* genes in each treatment (*p* > 0.05). The results showed that AOB population size was greater than AOA in the apple orchard, and DMPP inhibits the ammoxidation process by decreasing the abundance of AOB *amoA* gene.

#### Concentrations of Soil Mineral N (NH_4_^+^-N and NO_3_^–^-N)

**FIGURE 3:**
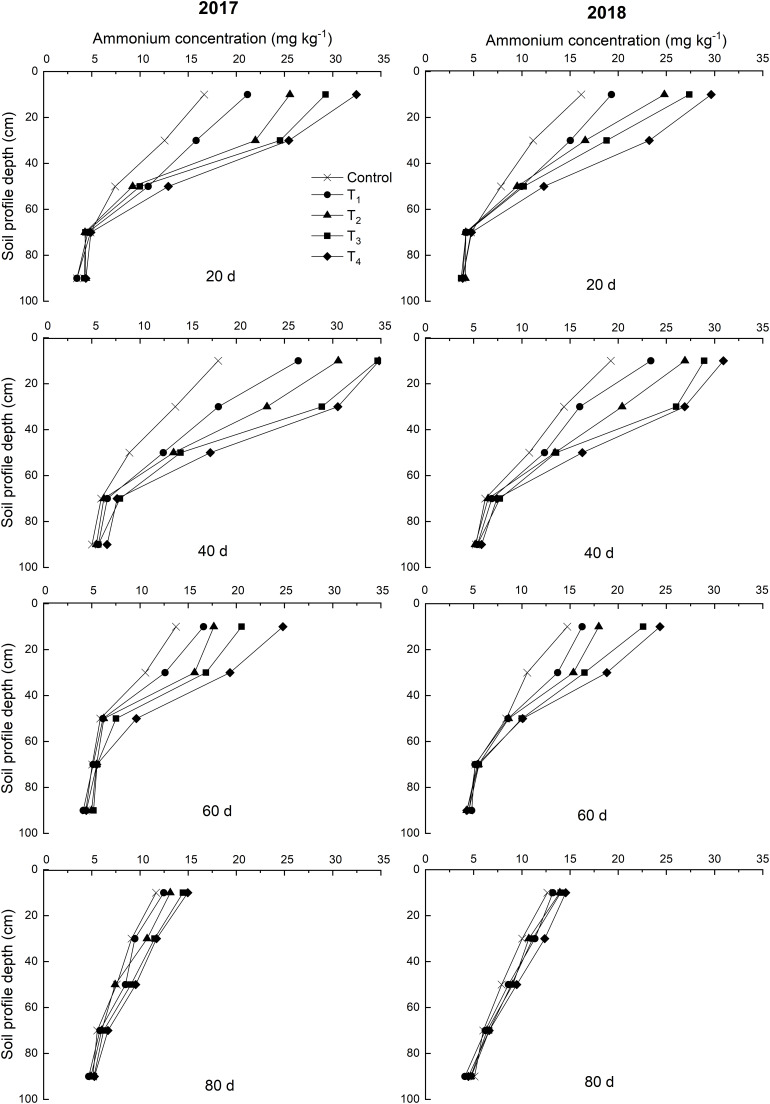
Effects of DMPP on the dynamic of NH_4_^+^-N concentration of soil in 2017 and 2018.

The trends of NH_4_^+^-N concentrations in each treatment were similar (Figure 3). In 2017 and 2018, the NH_4_^+^-N concentrations of the 0–60 cm soil layer were high, while those of the 60–100 cm soil layer were low, exhibiting a high to low distribution. The concentrations of NH_4_^+^-N in the soil increased within 60 days after DMPP application. The concentrations of NH_4_^+^-N increased as the DMPP dosage increased in the 0–60 cm soil layer, but no obvious differences were detected among the treatments in the 60–100 cm soil layer. No significant differences were detected in the NH_4_^+^-N concentrations among treatments after 60 days (*p* > 0.05; Figure 3).

**FIGURE 4:**
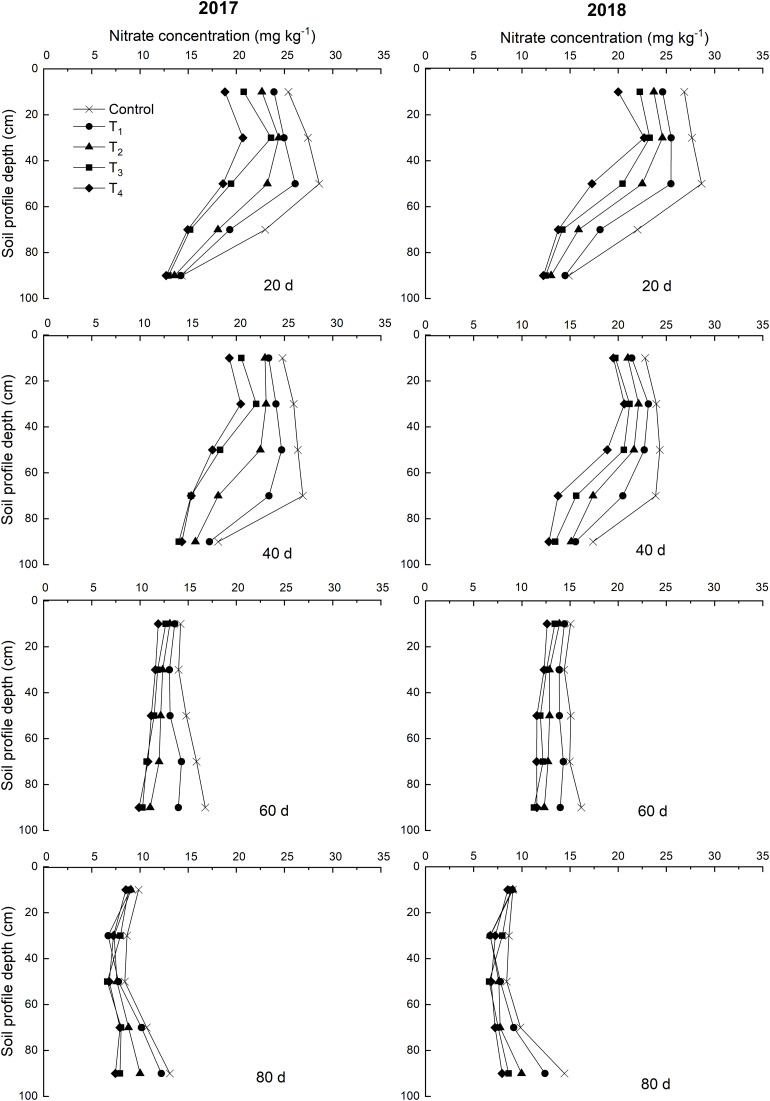
Effects of DMPP on the dynamic of NO_3_^–^-N concentration of soil in 2017 and 2018.

Over time, NO_3_^–^-N concentrations of control exhibited a vertical migration trend in 2017 and 2018 (Figure 4). The NO_3_^–^-N concentrations of the 0–60 cm soil layer decreased within 60 days after DMPP application and decreased as the DMPP dosage increased. No obvious differences were detected in the NO_3_^–^-N concentrations among treatments after 60 days. Therefore, DMPP treatment effectively inhibited the production of NO_3_^–^-N in the 0–60 cm soil layer and reduced the risk of NO_3_^–^-N vertical migration.

#### NH_3_ Volatilization Rates and Cumulative NH_3_ Volatilization

**FIGURE 5:**
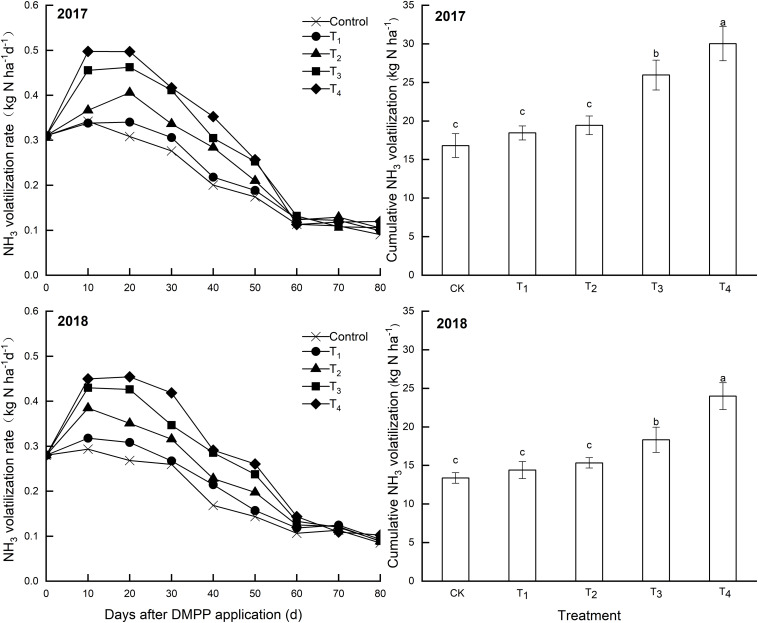
Effects of DMPP on the NH_3_ volatilization rate and cumulative NH_3_ volatilization in 2017 and 2018. The vertical bar indicates the standard deviation of three replications. Different letters indicate statistically significant differences (*P* < 0.05).

Over time, NH_3_ volatilization rates increased at first then decreased (Figure 5). Differences in the NH_3_ volatilization rates among treatments gradually decreased over time, and these differences were not obvious after 60 days. DMPP application increased NH_3_ volatilization rates and cumulative NH_3_ volatilization compared to the control, and both increased as the DMPP dosage increased (Figure 5). Compared to the control, the differences in cumulative NH_3_ volatilization were not significant (*p* > 0.05) when the DMPP dosage was low (T_1_ and T_2_), but that was significant (*p* < 0.05) when the DMPP dosage was high (T_3_ and T_4_).

### The Utilization, Residue and Loss of ^15^N

According to the roots distribution of dwarf apple, ^15^N within the 0–60 cm soil layers was considered to be soil ^15^N residue, while ^15^N in other soil layers means ^15^N loss. DMPP reduced the ^15^N utilization rate to varying degrees (Table 2). As the DMPP dosage increased, the^15^N utilization rate exhibited a downward trend. DMPP application increased the ^15^N residual and recovery rates, both of which reached the highest in T_2_. Compared to the control, the application of DMPP reduced ^15^N loss rate and the lowest value appeared in T_2_.

**TABLE 2:** Effects of DMPP on the utilization, residue and loss of ^15^N.

Year	Treatment	^15^N utilization rate (%)	^15^N residual rate (%)	^15^N loss rate (%)	^15^N recovery rate (%)
2017	Control	18.87 ± 1.32a	35.32 ± 2.02b	45.81 ± 2.48a	54.19 ± 2.48c
	T_1_	17.35 ± 1.32ab	38.42 ± 2.11b	44.24 ± 3.38ab	55.76 ± 3.38bc
	T_2_	16.55 ± 1.10b	45.06 ± 1.74a	38.39 ± 1.41c	61.61 ± 1.41a
	T_3_	15.36 ± 1.11b	44.95 ± 1.94a	39.69 ± 2.12c	60.31 ± 2.12a
	T_4_	15.30 ± 0.78b	43.56 ± 1.43a	41.13 ± 1.81bc	58.87 ± 1.81ab
2018	Control	20.52 ± 1.97a	37.63 ± 2.08c	41.85 ± 3.14a	58.15 ± 3.14b
	T_1_	18.52 ± 1.35ab	41.15 ± 2.00b	40.34 ± 2.54ab	59.66 ± 2.54ab
	T_2_	17.04 ± 1.13b	46.35 ± 1.80a	36.60 ± 1.44b	63.40 ± 1.44a
	T_3_	16.69 ± 1.35b	44.75 ± 1.47ab	38.55 ± 2.80ab	61.45 ± 2.80ab
	T_4_	16.13 ± 0.81b	43.07 ± 2.17ab	40.80 ± 2.41ab	59.20 ± 2.41ab

*Data are presented as the mean ± SD of three replicates. Different letters within a column indicate statistically significant differences between the means (*P* < 0.05).*

### Plant Organ Ndff and N Content

**FIGURE 6:**
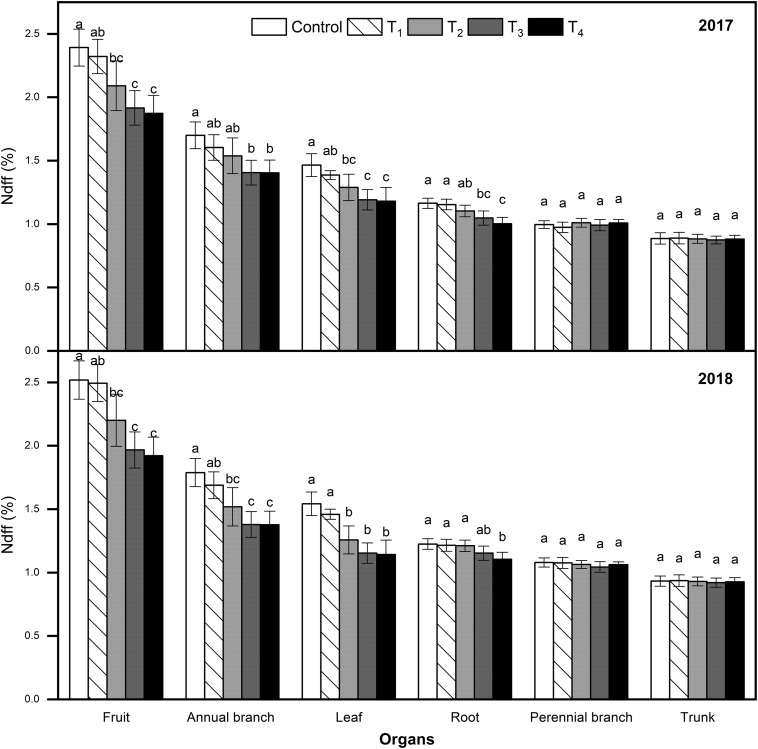
Effects of DMPP on Ndff value at the fruit maturity stage in 2017 and 2018. The vertical bar indicates the standard deviation of three replications. Different letters indicate statistically significant differences (*P* < 0.05).

Ndff refers to the ^15^N contribution rate absorbed from fertilizer and distributed by plant organs relative to the total N of plant organs, and reflects the ability of plant organs to absorb and regulate ^15^N fertilizer. The Ndff of organs subjected to different treatments were consistent at the fruit maturity stage across both years (Figure 6). In each treatment, the Ndff values were ordered as follows: fruits > annual branches > leaves > roots > perennial branches > trunk. Compared to the control, DMPP application reduced the Ndff of fruits, annual branches, leaves, and roots, which decreased as the DMPP dosage increased. No significant differences were detected on the Ndff of perennial branches and trunk among different treatments (*p* > 0.05). Results revealed that fruits absorbed and regulated ^15^N the most at the fruit maturity stage, while the annual branches and leaves also exhibited strong competitiveness. DMPP application reduced the ability of newborn organs to absorb and regulate ^15^N.

Accumulated ^15^N in the whole plant and fruits decreased gradually as the DMPP dosage increased (Table 3). Compared to the control, fruit ^15^N accumulation with DMPP application decreased by 10.53%–26.32% and 5.26%–15.79% in 2017 and 2018, respectively. Meanwhile, N contents in leaves and fruits also decreased gradually as the DMPP dosage increased (Table 3).

**TABLE 3:** Effects of DMPP on plant C–N nutrition.

Year	Treatment	^13^C accumulation in fruit (g plant^–1^)	^15^N accumulation		N content	
			Whole plant (g plant^–1^)	Fruit (g plant^–1^)	Leaf (g kg^–1^ DW)	Fruit (g kg^–1^ DW)
2017	Control	0.15 ± 0.01c	0.87 ± 0.06a	0.19 ± 0.01a	26.58 ± 1.03a	2.72 ± 0.08a
	T_1_	0.17 ± 0.01bc	0.79 ± 0.06ab	0.17 ± 0.01ab	26.06 ± 0.95ab	2.61 ± 0.05ab
	T_2_	0.20 ± 0.02a	0.76 ± 0.05b	0.17 ± 0.01ab	24.75 ± 0.83bc	2.50 ± 0.11bc
	T_3_	0.18 ± 0.01ab	0.71 ± 0.05b	0.15 ± 0.02bc	24.02 ± 0.50c	2.44 ± 0.06c
	T_4_	0.16 ± 0.01bc	0.70 ± 0.04b	0.14 ± 0.01c	23.63 ± 0.48c	2.43 ± 0.05c
2018	Control	0.16 ± 0.01b	0.94 ± 0.09a	0.19 ± 0.01a	26.81 ± 0.95a	2.75 ± 0.11a
	T_1_	0.18 ± 0.01b	0.85 ± 0.06ab	0.18 ± 0.01ab	26.22 ± 0.96ab	2.63 ± 0.07ab
	T_2_	0.20 ± 0.01a	0.78 ± 0.05b	0.17 ± 0.01abc	25.28 ± 0.85b	2.52 ± 0.11b
	T_3_	0.18 ± 0.01ab	0.77 ± 0.06b	0.16 ± 0.01bc	25.16 ± 0.51b	2.50 ± 0.07b
	T_4_	0.18 ± 0.02b	0.74 ± 0.04b	0.16 ± 0.02c	25.09 ± 0.24b	2.48 ± 0.17b

*Data are presented as the mean ± SD of three replicates. Different letters within a column indicate statistically significant differences between the means (*P* < 0.05).*

### ^13^C Distribution Rate and ^13^C Accumulation in Fruits

**FIGURE 7:**
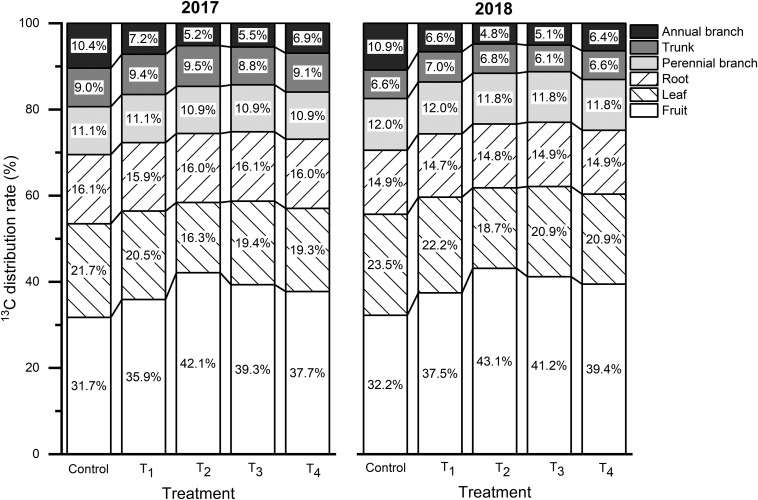
Effects of DMPP on the ^13^C distribution rate at the fruit maturity stage in 2017 and 2018 (^13^C distribution rate refers to the ratio of the ^13^C content of each organ to the amount of net ^13^C absorbed by the plant).

The proportion of ^13^C assimilates assigned to each organ is related to its competitive ability, which refers to the ability to absorb ^13^C from the leaves of active metabolic and growth organs. The ^13^C distribution rates for each treatment were consistent across both years, among which fruits had the highest value followed by leaves, roots, perennial branches, and trunk (Figure 7). DMPP increased the ^13^C distribution rate in fruits, which increased first and then decreased as the DMPP dosage increased. The highest ^13^C distribution rate in fruits appeared in T_2_, and the value increased by 10.36% and 10.87% compared to the control in 2017 and 2018, respectively. With an increasing of DMPP application rate, the ^13^C distribution rate in leaves and annual branches initially decreased and then increased, and the lowest value appeared in T_2_. No significant effect was observed on the ^13^C distribution rate in storage organs (roots, perennial branches, and trunk) (*p* > 0.05). Therefore, DMPP improved the competitiveness of fruits with respect to ^13^C and promoted ^13^C transportation from the vegetative organs (leaves and annual branches) to the fruits (Figure 7). Additionally, DMPP increased the ^13^C accumulation in fruits, and the highest value appeared in T_2_ treatment (Table 3).

### Fruit Yield and Quality

No significant effect was observed on fruit yield after DMPP application (*p* > 0.05; Table 4). However, DMPP had a positive effect on fruit quality. With an increasing of DMPP application rate, the anthocyanin contents of apple peels increased at first then decreased (Table 4). Compared to the control, the anthocyanin contents of T_2_ were the highest, which increased by 50.06% and 49.58% in 2017 and 2018, respectively. The trends of soluble sugar contents were consistent with anthocyanin contents. For titratable acids contents in fruits, the value decreased as the DMPP dosage increased. The sugar-acid ratio of T_2_ was the highest, and was 32.38% and 34.45% higher compared to the control in 2017 and 2018, respectively. Overall, appropriate DMPP application rate significantly improved fruit quality.

**TABLE 4:** Effects of DMPP on fruit yield and quality at the fruit maturity stage.

Year	Treatment	Fruit yield (kg plant^–1^)	Anthocyanin (mg 100 g^–1^ FW)	Soluble sugar (%)	Titratable acids (%)	Sugar/acid
2017	Control	25.76 ± 1.97a	17.16 ± 1.05^c^	12.05 ± 0.85b	0.47 ± 0.01a	25.85 ± 2.19^c^
	T_1_	25.41 ± 1.21a	19.85 ± 1.54b	13.26 ± 0.59ab	0.46 ± 0.02a	28.84 ± 1.80bc
	T_2_	25.96 ± 1.39a	25.75 ± 1.05a	14.70 ± 0.73a	0.43 ± 0.01b	34.22 ± 0.89a
	T_3_	25.87 ± 1.09a	24.79 ± 1.37a	13.91 ± 1.18a	0.42 ± 0.02b	32.66 ± 2.03a
	T_4_	25.40 ± 1.06a	23.71 ± 1.31a	13.07 ± 1.12ab	0.42 ± 0.01b	31.25 ± 2.42ab
2018	Control	28.33 ± 2.17a	17.87 ± 1.15b	12.34 ± 0.89b	0.46 ± 0.01a	27.20 ± 2.61b
	T_1_	28.45 ± 1.33a	20.83 ± 1.70b	13.60 ± 0.62ab	0.45 ± 0.02ab	30.45 ± 2.04b
	T_2_	28.56 ± 1.53a	26.73 ± 1.75a	15.12 ± 0.77a	0.41 ± 0.02bc	36.57 ± 0.44a
	T_3_	27.96 ± 1.20a	25.98 ± 1.51a	14.29 ± 1.25ab	0.41 ± 0.02bc	34.90 ± 2.15a
	T_4_	27.94 ± 1.16a	24.79 ± 1.95a	13.64 ± 1.42ab	0.39 ± 0.03^c^	34.89 ± 2.86a

*Data are presented as the mean ± SD of three replicates. Different letters within a column indicate statistically significant differences between the means (*P* < 0.05).*

## Discussion

### Effects of DMPP on Soil N Transformation and N Loss

Li et al. (2008) found that DMPP enhanced NH_4_^+^-N concentrations but reduced NO_3_^–^-N concentrations in the leachate and soil, as well as decreased the AOB population and soil nitrate reductase activities. This study found that DMPP inhibited the ammoxidation process by decreasing the abundance of AOB *amoA* gene. DMPP increased the abundance of AOA *amoA* gene, which is consistent with the results of Kleineidam et al. (2011). This may be related to DMPP changing the soil pH and microbial community structure (Li et al., 2011; Cao et al., 2018). The NH_4_^+^-N concentrations of the 0–60 cm soil layer were higher than that in the 60–100 cm soil layer, exhibiting a high to low distribution. This result was mainly due to the strong NH_4_^+^-N adsorption abilities of soil organic matter and colloidal particles; however, NH_4_^+^-N adsorption generally does not occur during vertical migration. Compared to the control, DMPP application decreased the concentration of NO_3_^–^-N and its vertical migration. Therefore, it was determined that DMPP could be used as an effective nitrification inhibitor to control ammonium oxidation and decrease soil NO_3_^–^-N concentration and its vertical migration, thereby minimizing shallow groundwater pollution risk. These findings are consistent with the results of Yu et al. (2007).

Nitrification inhibitors effectively prevent the occurrence of nitrification reactions. The mineralization of soil N is also enhanced by soil microorganisms, which results in the maintenance of soil ammonia N concentrations at higher levels. Therefore, the ammonia concentration gradient at the soil air interface is large, the ammonia diffusion ability is strong, and the rate of ammonia gas runaway is fast (Pinheiro et al., 2018). In this study, DMPP application increased NH_3_ volatilization rates and cumulative NH_3_ volatilization, and both increased as the DMPP dosage increased. Cumulative NH_3_ volatilization at high DMPP dosages was significantly different compared to the control. Overall, results revealed that the risk of soil ammonia volatilization significantly increased after DMPP reached a certain dosage. The prevention and control measures of ammonia volatilization after DMPP application need further study.

Previous studies found that DMPP increased fertilizer N and soil N recovery and decreased N runoff loss, which was beneficial to the ecological environment (Li et al., 2008; Yu et al., 2015; Alonso-Ayuso et al., 2016). Quemada et al. (2013) conducted a meta-analysis on irrigated agricultural systems and found that the use of nitrification inhibitors reduced nitrate leaching by 27% compared to conventional fertilizers. We found that DMPP reduced ^15^N loss rates, as well as increased ^15^N residual and recovery rates. DMPP application reduced ^15^N utilization rates but increased ^15^N residual rates during the growing season. Over time, DMPP was beneficial to the maintenance of the soil N pool, sustainable soil N supply capacity, and soil N absorption and utilization by trees during the subsequent growing season. However, a previous study found that DMPP significantly increased urea-N loss, which was due to the abundant ^15^NH_4_^+^-N retention and absence of plants in the soil condition with a high pH (Xu et al., 2019). These findings suggest that different environmental parameters (e.g., moisture, temperature, soil texture, pH, and the quality and quantity of soil organic matter) are modulated by the climate and agricultural management strategies, which should be considered when applying nitrification inhibitors (Niu et al., 2018; Zhang et al., 2019).

### Effects of DMPP on Tree C–N Nutrition and Fruit Quality

During the later stage of apple fruit expansion, the nutrients absorbed by trees mainly supplied fruit development. If the autumn shoots grew too much during this time, it led to nutrient dispersion, which thereby affected fruit quality. In this study, DMPP reduced autumn shoot length compared to the control (Supplementary Table S2). As the DMPP dosage increased, autumn shoot length gradually decreased. The growth of autumn shoots of control was too large, and the excessive growth of autumn shoots consumed several nutrients, which led to limited reproductive growth and was not conducive to fruit development.

C and N metabolism is the most basic metabolic process during fruit growth and development. C metabolism serves as a C source and provides energy for N metabolism, while N metabolism provides enzymes and photosynthetic pigments for C metabolism. The degree of coordination between C and N metabolism and their transformation directly and indirectly affect fruit quality. The late growth stage of apple trees is the key time when fruits convert from N to C nutrients. However, high temperature and rainy weather during this time lead to large soil NO_3_^–^-N supplies, resulting in vigorous N metabolism in tree. In this study, fruit N contents of control were 2.72 and 2.75 g kg^–1^ in 2017 and 2018, respectively (Table 3), which were higher than the optimal N contents of high-quality apple fruits (Zhang et al., 2017). High fruit N could reduce the activity of sugar metabolism enzymes in fruits, decrease the strength of fruit sink, and affect the transportation of carbohydrates to fruits, thus detrimentally affecting fruit quality (Kühn et al., 2011; Sha et al., 2019; Wang et al., 2020a). Therefore, coordinating C–N nutrition would benefit and improve fruit quality. In this study, the ^13^C and ^15^N double isotope labeling technology results revealed that DMPP reduced the capacity of ^15^N absorption and regulation in fruits, decreased ^15^N accumulation in fruits and whole plant, and improved the distribution of ^13^C from vegetative organs to fruits. Martínez et al. (2017) found that DMPP increased strawberry antioxidant compound contents, including vitamin C and total phenolics, and increased fruit quality. Yu et al. (2018) also reported that DMPP could improve pakchoi cabbage quality by regulating N transformation and heavy metal absorption. Consistent with previous results, we found that appropriate DMPP application dosages significantly improved fruit anthocyanin contents, soluble sugar contents, and sugar-acid ratios.

Nitrogen contents affect the distribution and accumulation of C in plant organs (Wang et al., 2020a). Our results found that higher leaves and fruits N contents in control and T_1_ inhibited C accumulation in fruits, which negatively affected fruit quality. Leaves and fruits N contents of T_3_ and T_4_ were lower, which resulted in deficient C metabolism precursors and affected the formation of fruit quality. T_2_ was more conducive to the transformation of C and N nutrition, C assimilate accumulation, and fruit quality improvement (Tables 3, 4 and Figures 6, 7).

## Conclusion

Appropriate DMPP application dosages could decrease the abundance of AOB *amoA* gene and the vertical migration of nitrate, thereby minimizing the risk of shallow groundwater pollution. Moreover, DMPP application reduced extravagant absorption of N in newborn organs, which in turn regulated the distribution and accumulation of C in fruits, so as to promote fruit quality. On the basis of our results, the application of 1 mg DMPP kg^–1^ soil during the later stage of fruit expansion could regulate soil N transfer and transformation, the C–N nutrition of trees, and effectively address the problems of N losses and fruit quality degradation caused by excessive N fertilizer application.

## Data Availability Statement

All datasets presented in this study are included in the article/Supplementary Material.

## Author Contributions

FW and YJ conceived and designed the experiments. FW, XX, XH, and ZJ performed the experiments. FW, SG, and ZZ wrote the manuscript. All authors have read and approved the final version of the manuscript.

## Conflict of Interest

The authors declare that the research was conducted in the absence of any commercial or financial relationships that could be construed as a potential conflict of interest.
