# Supplementary material for: Nitrification Inhibitor 3,4-Dimethylpyrazole Phosphate Application During the Later Stage of Apple Fruit Expansion Regulates Soil Mineral Nitrogen and Tree Carbon–Nitrogen Nutrition, and Improves Fruit Quality
Source: Front Plant Sci. 2020 Jun 3;11:764. doi: 10.3389/fpls.2020.00764 (PMC7285628; doi:10.3389/fpls.2020.00764)
Supplement: Supplementary file 1 [file Data_Sheet_1.docx]

Supplementary Material

**Supplementary Table S1** **|** Primer sets, reaction mixture compositions and thermocycling conditions used for target gene amplification in qPCR.

| Target gene | Primer Name | Primer sequences (5’-3’) | Contents of qPCR mixture (15 μl) | Length bp | qPCR programme | Amplification efficiency | R^2^ |
| --- | --- | --- | --- | --- | --- | --- | --- |
| AOA *amoA* | AOA-23F  AOA-616R | ATGGTCTGGCTWAGACG  GCCATCCATCTGTATGTCCA | 1 × SYBR green SYBR^®^  Ex Taq™ PCR mix (Takara, Dalian, China), 0.2 μM (each) primers, 2 μl template | 625 | **1 cycle**: 94°C 30s;  **40cycles**: 94°C 15s,  60°C 30s,72°C 45s,  80°C 10s signal detection;  melting curve | 94.8% | 0.997 |
| AOB *amoA* | AOB-1F  AOB-2R | GGGGTTTCTACTGGTGGT  CCCCTCKGSAAAGCCTTCTTC |  | 500 | **1 cycle**: 94°C 4 min;  **40cycles**: 94°C 45s,  55°C 30s,72°C 45s,  80°C 10s signal detection;  melting curve | 99.2% | 0.998 |

**Supplementary Table S2** **|** Effects of DMPP on autumn shoot length at the fruit maturity stage in 2017 and 2018.

| Year | Treatment | Autumn shoot length (cm) |
| --- | --- | --- |
| 2017 | Control | 30.40±2.13a |
|  | T_1_ | 21.39±1.47b |
|  | T_2_ | 14.70±1.33c |
|  | T_3_ | 12.08±1.13c |
|  | T_4_ | 9.31±0.88d |
| 2018 | Control | 34.14±2.47a |
|  | T_1_ | 23.71±1.70b |
|  | T_2_ | 15.97±1.53c |
|  | T_3_ | 12.93±1.30cd |
|  | T_4_ | 10.78±0.97d |

Data are presented as the mean ± SD of three replicates. Different letters within a column indicate statistically significant differences between the means (*P* < 0.05).
